# Impact of a diagnostic strategy for appendicitis in children with acute abdominal pain in primary care: study protocol for a hybrid type 1 cluster randomised controlled trial

**DOI:** 10.1186/s41512-026-00234-x

**Published:** 2026-08-13

**Authors:** Esmee M. Hogervorst, Roderick P. Venekamp, Grietje E. Knol-de Vries, Tamara N. Platteel, Roeland M. Watjer, Daan P. Hurkmans, Mattijs E. Numans, Guus C.G.H. Blok, Eline J.A.Q. Naber, Michiel R. de Boer, Karin M. Vermeulen, Meefa Hogenes, Eric Hiddink, P. Chris Tromp, Ramon R. Gorter, Albert Holvast, Jan B.F. Hulscher, Chantal M.C.R. Everts-Panman, Gera A. Welker, Sven J.G. Geelen, Arne R.M. van der Bilt, Elyne Deuring, Marjolein Y. Berger, Esen Doganer, Huibert Burger, Gea A. Holtman

**Affiliations:** 1 Department of Primary- and Long-term Care, Cure and Care in the Community Context (FOUR-C Research program), FA21, University of Groningen, University Medical Centre Groningen, PO Box 196, Groningen, 9700 AD The Netherlands; 2 Department of General Practice & Nursing Science, Julius Center for Health Sciences and Primary Care, University Medical Center Utrecht, Utrecht University, Utrecht, The Netherlands; 3 Department of Public Health and Primary Care, Leiden University Medical Center, Leiden, The Netherlands; 4 Department of Epidemiology, University Medical Center Groningen, Groningen, The Netherlands; 5 ExpertDoc B.V, Leiden, The Netherlands; 6 Health Base, Houten, the Netherlands; 7 Department of Surgery, Division of Pediatric Surgery, Amsterdam University Medical Center, Amsterdam, The Netherlands; 8 Department of Pediatrics, Martini Hospital Groningen, Groningen, The Netherlands; 9 Department of Pediatric Surgery, University Medical Center Groningen, Groningen, The Netherlands; 10 Department of Mother and Child, Wilhelmina Children’s Hospital, University Medical Center Utrecht, Utrecht, the Netherlands; 11 University Medical Center Staff Policy and Management Support, University Medical Center Groningen, Groningen, The Netherlands; 12 Research group Healthy Ageing, Allied Health Care and Nursing, Hanze University of Applied Sciences, Groningen, the Netherlands; 13 Department of Surgery, Ommelander Hospital Groningen, Scheemda, The Netherlands; 14 Department of Emergency Medicine, University Medical Center Groningen, Groningen, the Netherlands; 15 Department of Emergency Medicine, Ommelander Hospital Groningen, Scheemda, The Netherlands; 16 Stichting Kind & Zorg, Utrecht, The Netherlands

**Keywords:** Appendicitis, Primary care, Children, Clinical prediction rule, C-reactive protein

## Abstract

**Background:**

Children with acute abdominal pain pose a diagnostic challenge for general practitioners (GPs), as it can be difficult to distinguish appendicitis from self-limiting conditions due to overlapping symptoms. To support GPs, a diagnostic strategy for appendicitis was developed that integrates an externally validated prediction rule with C-reactive protein point-of-care testing (CRP POCT) and risk-based management advice. This study will compare the impact of this diagnostic strategy to usual care in children with acute abdominal pain in primary care in terms of: (i) clinical effectiveness, (ii) cost-effectiveness, and (iii) implementation potential.

**Methods:**

We will conduct a pragmatic, hybrid type 1 effectiveness-implementation, cluster randomised controlled trial in Dutch general practice. Children aged 4–18 years with acute (≤ 7 days) abdominal pain will be included. GP practices will be randomly allocated to either: (i) use of the diagnostic strategy for appendicitis, or (ii) usual care. In the intervention group, children are stratified into low, medium, or high risk for appendicitis based on a seven-item prediction rule including symptoms and signs. Management advice is tailored to appendicitis risk: safety netting for low-risk, immediate referral for high-risk, or CRP POCT for medium-risk (CRP < 10 mg/L: safety netting; CRP 10–50 mg/L: reassessment or immediate referral (based on GPs’ clinical judgement and parent/child preference); CRP ≥ 50 mg/L: immediate referral). The primary outcome is referral efficiency (proportion of non-referrals amongst patients with no appendicitis during 30 days follow-up). Secondary outcomes include safety, proportion of children with CRP POCT, proportion of children with planned reassessment, child anxiety, patient satisfaction, quality of life, and costs. A parallel process evaluation will assess implementation outcomes, including the reach, adoption, implementation, and maintenance of the diagnostic strategy. We aim to include 566 children without appendicitis to determine an improvement of efficiency from 88% to 95%.

**Discussion:**

We hypothesise that use of the diagnostic strategy will reduce referrals without increasing delayed diagnoses of appendicitis, compared to usual care in children presenting with acute abdominal pain in primary care. This may ultimately result in better patient outcomes, lower costs, and reduced health care resource use.

**Trial registration:**

ClinicalTrials.gov: NCT06762275 (registration date: 2024/12/03).

**Supplementary Information:**

The online version contains supplementary material available at 10.1186/s41512-026-00234-x.

## Background

Acute abdominal pain is a common reason for doctor consultations in children, accounting for nearly 10% of childhood visits in primary care [1]. More than 90% of these children have self-limiting conditions such as gastroenteritis or constipation [2, 3], whereas 5% have acute appendicitis, the prime reason for referral to secondary care in this population [2, 4–6]. Prompt recognition and referral of children with appendicitis by the general practitioner (GP) is crucial, as perforation occurs in 18% of cases [2] and is associated with high morbidity, including increased risk of postoperative complications (39%), pelvic abscess formation (53%), readmissions (14%) and prolonged hospitalisation [7–12]. Yet timely diagnosis of appendicitis is challenging in primary care, as early symptoms can be atypical and often overlap with those of benign conditions [5, 13, 14]. Moreover, current GP guidelines provide limited support, as they lack explicit referral criteria for appendicitis and do not recommend the use of C-reactive protein point-of-care testing (CRP POCT) due to insufficient evidence [4]. Consequently, 19% of children with appendicitis are not referred during the initial GP consultation, while 70% of those who are referred do not have appendicitis, which can lead to avoidable anxiety, healthcare utilisation and costs [2, 3, 15–18].

Although several prediction rules for identifying appendicitis in children exist in secondary care [13, 19], only one has been developed and validated in a primary care setting [20]. This rule, based on seven routinely available symptoms and signs, stratifies children into low-, medium-, or high-risk groups for appendicitis. External validation demonstrated good discrimination (c-statistic 0.90; 95% CI 0.89–0.92) and good calibration [20]. An additional study showed that CRP adds diagnostic value beyond symptoms and signs and may improve decision making in children with diagnostic uncertainty, corresponding with the medium-risk group of the prediction rule [21].

**Fig. 1:**
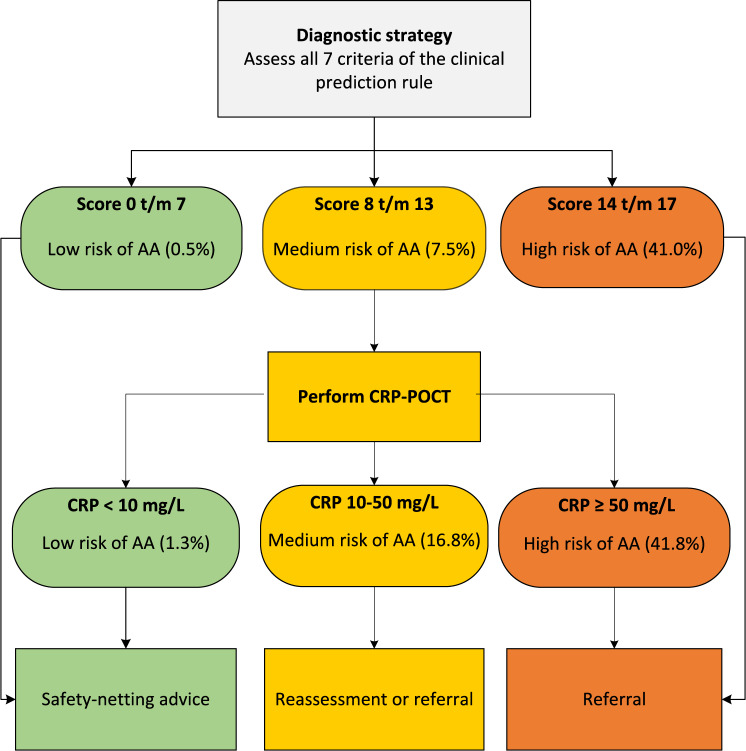
Diagnostic strategy for acute appendicitis, including the probability of appendicitis per risk group. AA, acute appendicitis; CRP, C-reactive protein; POCT, point-of-care testing

A low-cost diagnostic strategy combining the prediction rule with CRP POCT for medium-risk children (Fig. 1) could reduce referrals by providing risk-based management advice and is easy for GPs to use during consultations. However, its impact in everyday practice needs to be established before widespread implementation [22–25]. To address this knowledge gap, we designed a trial comparing the impact of the diagnostic strategy for appendicitis with usual care in children presenting with acute abdominal pain in primary care, in terms of: (i) clinical effectiveness, (ii) cost-effectiveness, and (iii) implementation potential.

## Methods

### Study design

**Fig. 2:**
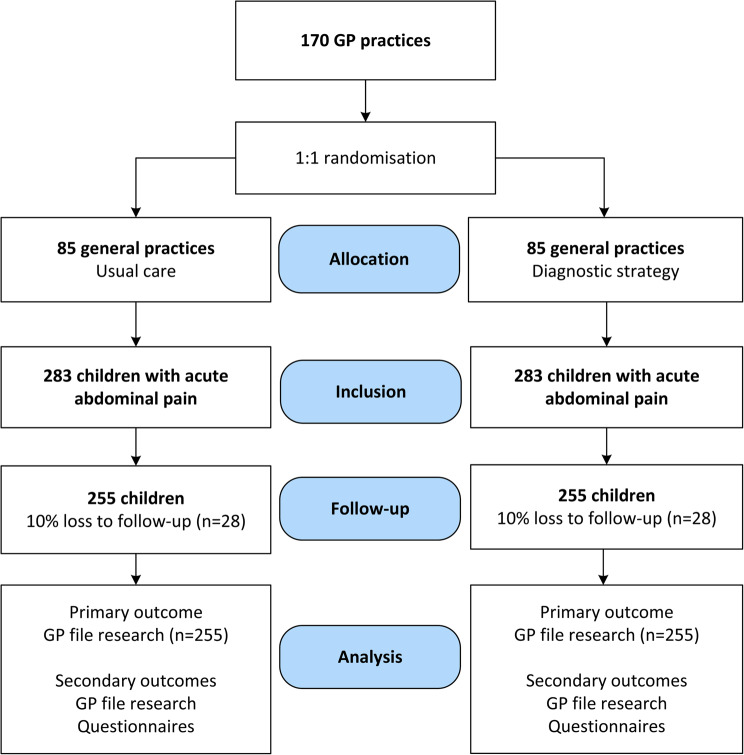
Flow-chart of the study design. GP, general practitioner

The ISAAK study (*Impact van een diagnostische Strategie voor Acute Appendicitis bij Kinderen*) is a pragmatic, hybrid type 1 effectiveness-implementation [26], superiority, cluster randomised controlled trial (RCT) in Dutch general practice, with GP practices as the unit of randomisation (Fig. 2).

Recruitment of GP practices started in November 2024, and the first child was enrolled in March 2025. The inclusion of children will continue until the required sample size is reached, which is expected in January 2028 (planned end date including 3 months follow-up: April 1st 2028).

The study protocol has been reviewed and approved by the Central Ethical Review Committee of the University Medical Center Groningen (UMCG) (METC number: 2022/399).

### Study population

Children aged 4 to 18 years, presenting to the GP with acute (onset ≤ 7 days) abdominal pain are eligible for inclusion. Children with prior appendectomy, abdominal pain caused by trauma or those who are pregnant are excluded.

### Recruitment

GP practices from three primary care networks (*Academic General Practitioner Development Network of UMCG*, *Julius General Practitioners Network of University Medical Center Utrecht* and *Extramural Leiden University Medical Center Academic Network*) and from the study team’s network are invited to participate by email. Participating GPs and GP trainees will recruit eligible children during office-hour consultations. Additionally, GP assistants will perform a monthly search of the primary care electronic health records (EHRs) of enlisted children using selected International Classification of Primary Care (ICPC) codes to identify and invite eligible children who have been missed by the GP (Supplementary 1).

**Fig. 3:**
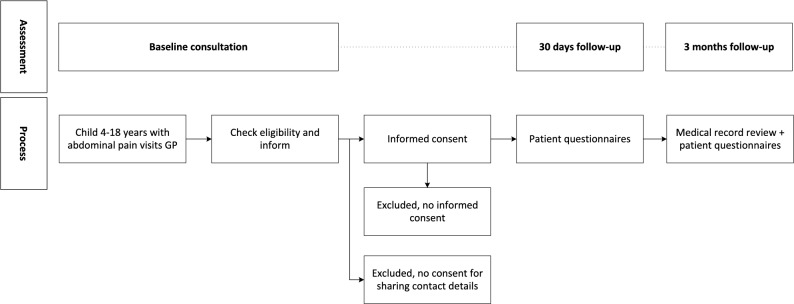
Recruitment process including data collection timeline. Anonymised baseline data (age, sex, date of baseline consultation, and randomisation group) will be collected for children who meet the inclusion criteria but do not provide consent to share contact details, or who object to participation after contact

Figure 3 outlines the recruitment process of children. During or after the consultation, the GP informs eligible children and/or parents about the study and asks consent to share contact and consultation data with the study team via an electronic case report form (CRF).

Upon retrieval of the contact and consultation data, the study team sends an email to the parent or child, including a patient information letter and informed consent form (Supplementary 2). Patients provide written consent for participation in the study, which includes completing questionnaires, and provide separate consent for collecting relevant data from the child’s EHR. If there is no response within seven days, the study team follows up by phone to provide further study details and to answer any questions.

### Randomisation, allocation and blinding

GP practices are randomly allocated to the intervention or control group using computer-generated 1:1 permuted-block randomisation with randomly varying block sizes, with stratification based on practice size (above or below 5000 patients). The randomisation is performed by an independent methodologist (H van der Worp, PhD). Blinding of GPs and patients is neither feasible nor appropriate in this pragmatic study [27]. Additionally, blinding of outcome assessors is not possible. However, data analysis will be conducted blinded to group allocation, meaning that the data analysts will be unaware of participant assignment until the analysis is complete. During the annual visits to participating general practices, the research team will verify any GP staffing changes. If a GP transfers from an intervention to a control practice or vice versa, their participation will be discontinued to avoid contamination.

### Control: usual care

GPs in the control group provide usual care at their own discretion. They are advised, but not obliged, to follow the NHG guideline ‘Abdominal pain in children’, which lacks specific referral criteria for appendicitis. The guideline advises suspicion of appendicitis in children with right lower quadrant pain, tenderness, and fever, and referral in case of peritonitis signs or abnormal bowel sounds. When in doubt, GPs are advised to plan a reassessment. CRP testing is not recommended due to lack of evidence. During a site initiation visit prior to the start of the study, the researchers present a summary of the NHG guideline to GPs. No additional education and/or materials are provided.

### Intervention: diagnostic strategy for appendicitis

GPs in the intervention group use a diagnostic strategy for appendicitis that integrates an externally validated seven-item prediction rule with CRP POCT and risk-based management advice. The prediction rule consists of routinely available signs and symptoms, each assigned a point score (Table 1).

**Table 1:** Clinical prediction rule for acute appendicitis

Predictor	Point score
Male sex	2
Nausea/vomiting	2
Pain duration < 24 h	1
Pain duration 24–48 h	2
Elevated temperature ≥ 37.3˚ C	1
Abnormal bowel sounds (absent, tinkling or high-pitched)	1
Right lower quadrant tenderness	5
Peritoneal irritation (abdominal rigidity, guarding, rebound tenderness and/or pain with jarring movements)	4

Based on the total score of the prediction rule, children are stratified into three risk groups: low (0–7 points), medium (8–13 points) and high risk (≥ 14 points) for appendicitis. A sensitivity and specificity for appendicitis of ≥ 95% correspond with the lower (7 points) and upper (14 points) cut-offs, respectively [20]. Management advice is tailored to appendicitis risk (Fig. 1): safety netting is advised for low-risk children, while immediate referral is recommended for those at high-risk. For children in the medium-risk group, GPs are instructed to perform CRP POCT. Based on the CRP value, the strategy recommends either safety netting (CRP < 10 mg/L), reassessment or immediate referral depending on the GP’s clinical judgment and parent/child preference (CRP 10–50 mg/L), or immediate referral (CRP ≥ 50 mg/L). The reassessment is considered a continuation of the initial consultation and includes a repeat clinical assessment and, if indicated, CRP POCT, and should take place later the same day or, if not feasible, the following morning. Cut-offs for CRP were derived from previous primary care research, where CRP ≥ 10 mg/L showed 0.87 (95% CI 0.77–0.94) sensitivity for appendicitis, increasing to 1.00 (95% CI 0.89-1.00) after 48 h, and CRP ≥ 50 mg/L demonstrated 0.94 (95% CI 0.93–0.96) specificity [21].

CRP POCT is performed in line with Dutch laboratory standards. For all participating practices, we will record both the availability and the type of CRP POCT device. Since all CRP POCT devices used in Dutch general practice have good accuracy and comparability [28–30], each practice will use its own device. GPs are instructed to follow the diagnostic strategy as much as possible, but are informed that they may deviate from the recommended management when deemed necessary. During the site initiation visit, the researchers give a brief presentation of the NHG guideline and the diagnostic strategy to GPs. Each GP also receives a pocket card containing the diagnostic strategy.

### Implementation strategies

As shown in previous research, diagnostic strategies are often not used or used incorrectly by GPs, which can reduce efficiency and increase failure rates [31–33]. We therefore complemented the diagnostic strategy with targeted implementation strategies to improve adherence [34–38]. Prior to study conduct, we identified barriers and facilitators through qualitative research, literature, and stakeholder input using the Consolidated Framework for Implementation Research (CFIR) (Supplementary 3) [39]. Based on these findings, we identified implementation strategies using the CFIR-Expert Recommendations for Implementing Change (ERIC) Matching Tool and developed these into study-specific strategies (Supplementary 4). The two main strategies are online training and a clinical decision tool integrated in the primary care EHR system, based on the ERIC strategies: developing educational materials, promoting adaptability and reminders for clinicians [40–44].

#### Online training

Before study initiation, GPs in the intervention group complete an online training on the diagnostic strategy. The training was developed during two expert panel sessions with three practicing GPs (MEN, NK, TNP), two paediatric surgeons (RRG, JBFH), a paediatrician (AH), two clinical epidemiologists (GAH, HB), a non-practicing GP, an emergency physician, and an educationalist. The first session involved a discussion about the goals, structure and content of the training. Based on the input, a draft version of the training was developed by EMH and tested by eight panel members. In the second session the draft was refined, resulting in a final version. All panel members tested and approved the online training before it was implemented.

The final 60-minute online training includes five modules on acute abdominal pain in children, use of the prediction rule and CRP POCT, and risk-based communication with parents and children (Supplementary 5). Each module includes a mix of text, figures, tables, questions, and videos of GP consultations with a child presenting with acute abdominal pain and their parent. Adherence to the online training is monitored via the training module’s registration data.

#### Clinical decision support tool integrated in primary care EHR system

Participating GPs receive an electronic clinical decision support tool integrating the diagnostic strategy into the primary care EHR system. The tool was developed in collaboration with software developers (ExpertDoc and Health Base). It is available in two formats, depending on the practice’s type of EHR system. Both versions were tested and approved by six academic and non-academic GPs from the project team.

For GP practices using Medicom as EHR system, the diagnostic strategy is directly integrated into the EHR. It appears in real time as a pop-up when an ICPC-code for gastrointestinal symptoms (D.xx) is entered for children aged 4 to 18 years, enabling the GP to record the presence of signs and symptoms, and CRP POCT value if needed, and receive tailored recommendations.

For non-Medicom practices, direct integration into the EHR was not feasible. Instead, GPs access the tool via a REDCap (Research Electronic Data Capture) link, either through a desktop application or the NHGDoc Classic button. This button, available in five Dutch EHR systems (CGM, OmniHIS, Promedico ASP, microHIS, Bricks), turns yellow when an ICPC D.xx code has been entered, directing the GP to the REDCap tool.

### Data collection

GPs will complete a baseline CRF for eligible children during the initial consultation, which contains the inclusion and exclusion criteria, contact details of the parent or child, and questions on whether CRP POCT or referral was performed, and why. For GPs in the intervention group, the diagnostic strategy is also included (Supplementary 6).

Medical record data will be collected for all participating children using a standardized form (Supplementary 7). Parents and/or children will complete electronic questionnaires on secondary outcomes via REDCap. Parents complete questionnaires for children ≤ 7 years. For children aged 8–15 years, both the child and parent complete questionnaires separately. Children ≥ 16 years complete all questionnaires independently, except for the cost questionnaire, which they fill out together with a parent.

Questionnaires are emailed at 30 days and 3 months after baseline. Automated reminders from REDCap are sent after 5 days and 10 days. If questionnaires remain incomplete after 10 days, the study team follows up by phone.

All study-related and participant information is stored securely at the study site on institutional servers of the University of Groningen and the University Medical Center Groningen, accessible only to authorized researchers.

### Effectiveness outcomes

Table 2 outlines the effectiveness outcomes.

**Table 2:** Effectiveness outcomes

Primary outcome	Measurement	Baseline	30 days	3 months
Referral efficiency	Medical records		x	
Secondary outcomes				
Safety	Medical records		x	
Proportion of CRP POCT^a^	Management data in CRF^a^ Medical records	x		
Proportion of planned reassessment	Management data in CRF^a^ Medical records	x		
Child anxiety	Dutch version of STAIC^a^		x	x
Parental or child satisfaction	Dutch version of P-MISS^a^		x	
Quality of life	EQ-5D-proxy (≤ 7 years), EQ-5D-Y (8–15 years), EQ-5D-3 L (≥ 16 years)^a^		x	x
Costs	Medical records Adjusted iPCQ^a^ and iMCQ^a^		x	x
Other (descriptive) outcomes				
(Adverse) health outcomes	Medical records			x

^a^Abbreviations defined below

*CRP* C-reactive protein, *POCT* point-of-care testing, *CRF* case report form, *STAIC* State-Trait Anxiety Inventory for Children, *P-MISS* Parental Medical Interview Satisfaction Scale, *EQ-5D* EuroQol 5D, *iPCQ* iMTA (Institute for Medical Technology Assessment) Productivity Cost Questionnaire, *iMCQ* iMTA Medical Consumption Questionnaire

#### Primary outcome

The primary outcome is referral efficiency, defined as the proportion of non-referrals in children without appendicitis during 30 days follow-up (Table 3). The diagnosis of appendicitis will be based on imaging, intraoperative findings, and/or histopathology as reported by the secondary care specialist. After 30-days of follow-up, the development of appendicitis is considered highly unlikely.

#### Secondary outcomes

#### Safety

Safety is defined as the proportion of children with appendicitis who are referred during the initial GP consultation or planned reassessment (Table 3).

#### Proportion of children with CRP POCT

The proportion of children in whom CRP POCT was performed during the initial GP consultation will be recorded for each randomised group.

#### Proportion of children with planned reassessment

The proportion of children for whom a planned reassessment was performed after the initial GP consultation will be recorded for each randomised group.

#### Child anxiety

Anxiety in children is assessed using the 20-item Dutch version of the State-Trait Anxiety Inventory for Children (STAIC) at 30 days and 3 months follow-up. The Dutch version of the STAIC (Zelfbeoordelingsvragenlijst voor Kinderen (ZBV-K) Versie Nu) measures self-perceived anxiety based on the state-anxiety subscale and is feasible for children ≥ 8 years. The Dutch version of the state-anxiety subscale has demonstrated good validity and reliability in children (Cronbach’s alpha 0.80 or higher) [45].

#### Parental or child satisfaction about baseline consultation

The satisfaction of parents (children 4–15 years) or children (16–18 years) with the GP consultation is assessed at 30 days follow-up using the Dutch version of the Parental Medical Interview Satisfaction Scale (P-MISS). Comprising 26 items, this questionnaire measures satisfaction regarding communication with parent and child, distress relief and adherence intent, rated on a five-point Likert scale (1 = strongly disagree to 5 = strongly agree) [46]. The P-MISS shows good construct validity and internal consistency (Cronbach’s alpha 0.86–0.95) [46, 47].

#### Child’s quality of life

Quality of life will be evaluated at 30 days and 3 months follow-up using the EuroQol 5D 3 L (EQ-5D-3 L), a generic instrument measuring health-related quality of life (HRQOL). The EQ-5D-3 L includes five domains (mobility, self-care, usual activities, pain or discomfort and anxiety or depression) that are each rated on a three-level severity scale (0 = no problems, 1 = some problems, 2 = extreme problems). The EQ-Visual Analogue Scale records the child’s overall health status with scores ranging from 0 to 100. Parents will complete the EQ-5D-3 L proxy for children aged ≤ 7 years. Children aged 8–15 years will complete the EQ-5D-youth (EQ-5D-Y) and children ≥ 16 years will complete the EQ-5D-3 L. The EQ-5D-Y is highly feasible for assessing HRQOL in children and adolescents with different health conditions [48–51]. Additionally, it has good convergent validity and fair to moderate test-retest reliability (test-retest agreement 70–99%) [49, 51].

#### Costs

Medical and societal costs will be assessed using adapted versions of the iMTA Productivity Cost Questionnaire (iPCQ) and the iMTA Medical Consumption Questionnaire (iMCQ), completed at 30 days and 3 months follow-up. Parents (for children ≤ 15 years) or parents and child (≥ 16 years) will report healthcare use, out-of-pocket expenses, and productivity losses (absence from work). Additionally, researchers will extract information regarding healthcare consumption from children’s medical records (e.g. CRP POCT, GP visits, referrals, hospital admissions, operative and non-operative management in hospital, including antibiotic therapy). All standard-of-care, including all types of healthcare usage, is included in the cost-effectiveness analysis. Medical equipment costs for CRP POCT will be calculated according to the Dutch costing manual, including depreciation, interest, and maintenance costs. Annualized equipment costs will be allocated per procedure based on the expected annual number of measurements on the machine.

The units measured will be monetised according to guidelines of the National Healthcare Institute of the Netherlands [52].

**Table 3:** Definitions of safety and efficiency

	Appendicitis +	Appendicitis -
Referred ^a^	a	b
Not referred	c	d

Definitions: Safety (sensitivity) = a / (a + c). Efficiency (specificity) = d / (b + d). The sample size calculation is based on the denominator of the efficiency outcome (b + d)

^a^ Referred at the initial GP consultation for acute abdominal pain or at planned reassessment

### Implementation outcomes

In addition to the aforementioned effectiveness outcomes, implementation outcomes include Reach, Adoption, Implementation and Maintenance. Reach is measured on both patient and GP (practice) level, while adoption, implementation and maintenance are measured at GP level in the intervention group. Quantitative data will be obtained from project records, medical records, and decision tool logs. Qualitative data will be obtained through semi-structured interviews with GPs in the intervention group during and after the trial. The interviews will explore GPs’ experiences with the diagnostic strategy, including barriers, facilitators, and reasons for its use or non-use in terms of adoption, implementation and maintenance, as well as their experiences with and reasons for using or not using the implementation strategies. We will use an inductive approach to thematic saturation [53], guided by sensitizing concepts derived from the literature. Qualitative data collection stops when thematic saturation is reached, defined as the point at which additional interviews no longer generate new information relevant to each implementation outcome.

#### Reach

Reach refers to the representativeness of participating children, GPs and GP practices. Outcome measures include the proportion and demographics of children exposed to the diagnostic strategy (from all eligible children), participating GPs (from all invited GPs), and participating GP practices (from all invited practices). Reasons for non-participation will also be documented.

#### Adoption

Adoption is the proportion of children per GP in the intervention group for whom the diagnostic strategy was used (from all eligible children seen by the GP).

#### Implementation

Implementation refers to adherence to both the intervention and implementation strategies. Outcomes for implementation include the proportion of children per GP in the intervention group for whom the diagnostic strategy was applied as intended (from all children for whom the strategy was used), and the proportion of GPs in the intervention group who used the two main implementation strategies.

#### Maintenance

Maintenance refers to sustained adoption and intervention adherence by GPs two years after inclusion. We will assess this quantitatively using the three-item Provider REport of Sustainment Scale (PRESS), which is a brief and pragmatic outcome measure on the continued use of an evidence-based practice, showing good validity and reliability (Cronbach’s alpha: 0.947) [54].

### Sample size considerations

The prediction rule at a cut-off of 14 points and a CRP value of ≥ 50 mg/l has a specificity of 97% and 94%, respectively [20, 21]. We therefore anticipate an improvement of efficiency from 88% (current care [2]) to 95% (the weighted mean of the aforementioned specificities). To detect the 7% difference in efficiency with 80% power at a two-sided 5% significance level, a cluster size of 3 and an intraclass correlation of 0.01 [55], the required sample size is 510 children without appendicitis (255 per arm). Adjusting for an anticipated 10% loss to follow-up, we aim to include a total of 566 children without appendicitis (283 per arm). Accordingly, we will include all children assessed for appendicitis, including those with appendicitis, until the target sample of 566 children without appendicitis has been reached. Although children with appendicitis do not contribute to the formal sample size calculation for our trial, which is powered for the primary outcome (efficiency), they will contribute to the assessment of the secondary outcome (safety). With an average of two to six children presenting during office hours per general practice in two years [2, 56, 57], we aim to recruit at least 170 GP practices with a mean patient population (85 per arm).

### Data analysis

#### Clinical effectiveness

We will present descriptive statistics to summarise and compare baseline characteristics of GPs and children between the intervention and control groups. The primary analysis will be on an intention-to-treat basis, i.e. based on the randomised group allocation of the children’s GP practice, regardless of the actual care received. Additionally, a per protocol analysis will be performed, including only children in the intervention group who received the diagnostic strategy and CRP POCT as intended, and children in the control group who did not undergo CRP-testing.

Analyses will be carried out on the individual patient level. Dichotomous outcomes (proportions) will be analysed using logistic mixed-effect regression models with fixed effects for group allocation and practice size, and a random effect for practice to account for clustering of patients within GP practices. Continuous outcomes will be analysed using linear mixed effects regression models with the same approach. When outcomes are measured repeatedly, an additional random effect for patient will be included to account for within-patient correlation, along with fixed effects for time and, if appropriate, its interaction with allocation. If necessary, fixed effects for clinically meaningful differences in baseline characteristics will be included in supplementary analyses. Exploratory analyses will be performed to assess subgroup differences in effectiveness by age and sex through inclusion of the corresponding treatment-by-subgroup interaction terms in the regression models.

Estimates of intervention effects ((adjusted) mean differences or ORs, as appropriate) are presented with 95% confidence intervals (CIs). Adverse health outcomes (e.g. complications in appendicitis cases and non-appendicitis cases, missed other emergency diagnoses) will be summarised descriptively, without further statistical analysis.

#### Cost-effectiveness

Cost-effectiveness will be calculated from both a societal (including productivity loss) and a health care perspective with a time horizon of 30 days. Secondary analyses will address a 3-month time horizon. Both cost-effectiveness and cost-utility analyses will be performed to compare the ratio of costs and effects between arms. In the cost-effectiveness analysis, incremental cost effectiveness ratios will be calculated, using efficiency as effect parameter. Cost-utility will be calculated based on the EQ-5D-(Y)-3 L, by which we will determine the added costs per quality-adjusted life year gained. For the EQ-5D-3 L proxy and EQ-5D-Y, utility scores will be derived using the Dutch value set [58]. For the EQ-5D-3 L, the Dolan algorithm will be applied [59]. Bootstrap re-sampling will be performed in order to estimate CIs. Additionally, based on the bootstrap replications, cost-effectiveness and cost-utility planes will be plotted, along with cost effectiveness acceptability curves (CEACs).

#### Process evaluation

We will calculate descriptive statistics for the quantitative items of the RE-AIM outcome measures. Thematic content analysis will be performed for the semi-structured interviews on adoption, implementation and maintenance. To understand why the implementation strategies did or did not work, we will construct causal pathway diagrams that map the mechanisms, determinants, and proximal and distal outcomes of each implementation strategy [60].

### Patient and public involvement

Patient and public involvement is embedded throughout the project and documented in the patient involvement matrix (Supplementary 8). We collaborate with the Dutch Child and Care Foundation (*Stichting Kind en Zorg*), which provided advice on the grant proposal, patient information letters, recruitment strategy and qualitative research. They will also support dissemination of the study results to the public. Additionally, a patient panel (two parents and one child with a history of acute abdominal pain) advised on the information letter, decision-making tool and recruitment strategy, and will contribute to the sharing of results.

## Discussion

To our knowledge, this study is the first to evaluate the impact of a diagnostic strategy for appendicitis that combines an externally validated prediction rule and CRP POCT with risk-based management advice in children with acute abdominal pain in primary care. By providing GPs with more guidance in their decision making, the diagnostic strategy could reduce uncertainty. We hypothesise that this will, in turn, help lower referral rates without compromising safety, thereby improving patient outcomes (e.g. quality of life, anxiety, satisfaction) and reducing healthcare costs [61, 62].

To ensure clinical relevance and validity, diagnostic strategies must be developed and tested in the setting where they are intended to be used [63]. This typically follows sequential phases: development, accuracy assessment, evaluation of clinical and cost-effectiveness, and implementation [23–25]. To date, prediction rules for appendicitis have predominantly been developed in secondary care, with few being evaluated for impact in RCTs [19, 64]. However, these rules are not suitable for use in primary care, where children present earlier and with different symptom profiles, laboratory tests (e.g. white blood cell counts) are not readily available at the point of care, and diagnostic decisions focus on referral rather than imaging or surgery [65, 66]. Therefore, our group developed a prediction rule tailored to primary care, which forms the basis of the diagnostic strategy assessed in this trial [20]. The CRP cut-offs used in the diagnostic strategy (10 and 50 mg/L) are based on previous research in primary care [21] and supported by evidence from secondary care, where CRP ≥ 50 mg/L showed the highest specificity of all blood tests (0.87; 95% CI 0.80–0.91) [67]. However, implementation of CRP POCT will also depend on the acceptability and perceived burden of additional blood testing among children and their parents, which will be explored in the qualitative study.

Impact trials of diagnostic prediction rules are rarely conducted in primary care, largely due to their complexity, costs, and duration [22]. Nevertheless, we opted for an RCT to generate the highest possible level of evidence [68]. An additional strength of our study is its pragmatic hybrid type 1 design, which enables simultaneous evaluation of effectiveness and implementation outcomes, supporting direct translation of the study findings into daily practice [69–72]. Further strengths include the use of implementation strategies to optimise adherence, as well as a three-month follow-up period that allows comprehensive assessment of healthcare resource use. We selected referral efficiency as the primary outcome, in line with recommendations by Reilly et al. [22]. Safety was defined as a secondary outcome, as using it as a co-primary outcome would have required an unfeasibly large number of children with appendicitis. Therefore, although the results will provide supportive evidence regarding the safety of the intervention, any observed differences between the arms in this outcome must be interpreted with caution due to the lack of adequate power. There could be some misclassification in diagnosing appendicitis, as there are rare cases of spontaneously resolving appendicitis that might be missed [73]. However, since these cases would not require a referral for surgery, this misclassification will not influence the clinical relevance of our outcome.

Still, our study has some limitations. First, although the cluster design reduces potential contamination between GPs [74, 75], it can introduce selection bias if GPs in the intervention arm include children with different characteristics (e.g. more severe symptoms) than those in the control group [76]. To address this, missed eligible children are identified retrospectively via GP registries, and their characteristics compared with those of included children for both groups, with adjusted analyses conducted if baseline differences are observed. Second, GPs and patients are not blinded, which may lead to bias. However, we consider this bias to be limited, as pragmatic trials assess overall effects on the care pathway rather than isolated intervention effects [27]. Third, children presenting at out-of-hours primary care service are not included because of logistical constraints related to the inclusion of GPs and children at these services, particularly the training of an unfeasibly large number of GPs and the reviewing of medical records at numerous individual practices within the service’s catchment area. This may limit generalizability of findings to out-of-hours settings due to potential differences in urgency and severity of appendicitis.

In summary, this study will evaluate whether a diagnostic strategy for appendicitis in children presenting to their GP with acute abdominal pain can reduce referrals to secondary care without increasing delayed diagnoses, compared to usual care. If our hypothesis is confirmed, the findings may support its adoption in routine practice, ultimately contributing to more efficient care and better outcomes for these children.

## Supplementary Information

Supplementary Material 1.

## Data Availability

We will make all metadata (including the study protocol, analysis syntaxes, data management plan, codebook, and related files) available through the Groningen Data Catalogue. The pseudonymised dataset will be accessible upon reasonable request and pending approval by the principal investigator (GAH) and all consortium partners.
