# Supplementary material for: Impact of a diagnostic strategy for appendicitis in children with acute abdominal pain in primary care: study protocol for a hybrid type 1 cluster randomised controlled trial
Source: Diagn Progn Res. 2026 Aug 13;10:22. doi: 10.1186/s41512-026-00234-x (PMC13474870; doi:10.1186/s41512-026-00234-x)
Supplement: Supplementary file 1 [file 41512_2026_234_MOESM1_ESM.docx]

**Supplementary 1. International Classification of Primary Care codes for retrospective search in general practice registry**

| **International Classification of Primary Care code** | **Description** |
| --- | --- |
| D01 | Generalized abdominal pain/cramps |
| D02 | Epigastric abdominal pain |
| D03 | Acid reflux |
| D06 | Other localized abdominal pain |
| D09 | Nausea |
| D10 | Vomiting |
| D11 | Diarrhoea |
| D12 | Constipation |
| D25 | Change of circumference/abdominal distension |
| D70 | Infectious diarrhoea, dysentery |
| D73 | Presumed gastro-intestinal infection |
| D88 | Appendicitis |
| D93 | Irritable bowel syndrome |

**Supplementary 2. Informed consent forms**

**Supplementary 3. Expected facilitators and barriers to the decision tool for general practitioners categorized by CFIR domain**

| **Domain** | **Facilitators** | **Barriers** |
| --- | --- | --- |
| **1. Innovation *Clinical decision tool*** A. Source B. Evidence-Base C. Relative advantage  D. Adaptability E. Trialability F. Complexity G. Design H. Cost | - Decision tools are often included in clinical guidelines (1B)^a^  - Trust in the reasoning and evidence behind the decision tool is available to GPs (1B)^1^  - Decision tools provide structure and guidance (1C)^a^  - Less subjectivity (1C)^a^  - The decision tool covers key medical information (1C)^a^  - The decision tool complements clinical judgement (1C)^a^  - The decision tool can facilitate the diagnostic process (1C)^a^  - The decision tool supports clearer explanations to parents based on objective parameters (1C)^a^  - The decision tool is applicable when history-taking with the child is not possible (1C)^a^  - GPs can deviate from the tool when needed (1D)^a^  - Clear and structured workflow (1G)^a^ | - Publications in GP journals are essential (1B)^a^  - Limited knowledge about interpretation of CRP in children with acute abdominal pain in primary care (1B)^a^  - Lack of evidence on the indication of CRP in children with acute abdominal pain in primary care (1B)^a^  - Few issues with current practice (1C)^a^  - Unclear clinical added value of the decision tool (1C)^a^  - Decision tool only adds value in cases of diagnostic uncertainty (1C)^a^  - Unclear terminology in the decision tool (e.g. abnormal bowel sounds) (1F)^a^  - Costs of purchasing and using the decision tool after implementation (1H) |
| **2. Outer setting *Society***  A. Critical Incidents B. Local Attitudes C. Local Conditions  D. Partnerships & Connections  E. Policies & Laws F. Financing G. External Pressure | Fewer referrals of children without appendicitis, resulting in more efficient care, lower healthcare costs, and reduced burden on secondary care (2D) | - CRP is often performed because secondary care requests this (2D)^a^  - Trust arises only once the decision tool is included in the NHG guideline (2E)^a^ |
| **3. Inner setting**  ***General practice***  A. Structural Characteristics B. Relational Connections C. Communications D. Culture E. Tension for Change F. Compatibility G. Relative Priority H. Incentive Systems I. Mission Alignment J. Available Resources K. Access to Knowledge & Information | - Easy integration into the primary care EHR system (3A)^a^  - Follow-up allows room for GP’s own interpretation (3A)^a^  - Safety-netting advice is clear (3C)^a^  - Currently no alternative to clinical judgement (3E)^a^  - NHG guideline provides limited guidance (3E)^a^  - Currently relatively many unnecessary referrals (3E)^a^  - Fingerprick CRP is less traumatic for the child than a hospital visit (3E)^a^  - Seven symptoms in the decision tool align with GP’s gut feeling (3F)^a^  - The diagnostic strategy should be integrated into the GP’s workflow (3F)^1^  - Recognizing appendicitis clinically is difficult due to low prevalence (3I)^a^ | - Integration into the primary care EHR system feels patronizing, limiting the GP’s autonomy (3A)^a^  - GPs’ decision-making is intuitive rather than analytical (3F)^1,2^  - Probabilistic knowledge without direct recommendations is difficult to apply in decision-making (3F)^1^ |
| **4. Individuals**  A. Need B. Capability C. Opportunity D. Motivation | - Possibly fewer referrals of children without appendicitis, improving patient outcomes such as satisfaction, quality of life and anxiety (4A)  - Possibly faster referral of children with appendicitis, reducing the risk of morbidity from perforation (4A)  - The diagnostic strategy takes little time to use in practice (4C)^a^ | - No need for a new tool given the low prevalence of appendicitis in children (4A)^a^  - CRP POCT can be painful for children (4A)^3^ |

Abbreviations: GP, general practitioner; CRP, C-reactive protein; NHG, Nederlands Huisartsen Genootschap; EHR, electronic health record; POCT, point-of-care testing

^a^ Based on semi-structured interviews with 10 general practitioners about the barriers and facilitators of the diagnostic strategy.

^1^ Kappen TH, van Loon K, Kappen MAM, van Wolfswinkel L, Vergouwe Y, van Klei WA, et al. Barriers and facilitators perceived by physicians when using prediction models in practice. J Clin Epidemiol 2016 Feb:70:136-45.
^2^ Mulder LJ, Ansems SM, Berger MY, Blok GC, Holtman GA. GPs' perspectives on diagnostic testing in children with persistent non-specific symptoms: a qualitative study. Br J Gen Pract. 2025;75(751):e105-e12.
^3^ Schot MJC, Broekhuizen BDL, Cals JWL, Brussee E, de Wit NJ, Verheij TJM, de Groot E. C-reactive protein point-of-care testing in children with cough: qualitative study of GPs’ perceptions. BJGP Open. 2017 Oct 27;1(4):bjgpopen17X101193.

**Supplementary 4. Implementation strategies based on the CFIR-ERIC Matching tool**

| **Period** | **CFIR factors** | **ERIC strategies** | **Application in this study** |
| --- | --- | --- | --- |
| Before trial (Apr 2024-Dec 2024) | **1B, 1C, 1D, 1F, 4A** | Develop educational materials | Development of an online training for GPs in the intervention group to complete before the kick-off visit |
| Before trial (Apr 2024-Dec 2024) | **1C, 1D, 1F, 1G, 3A, 3E, 3F** | Promote adaptability Remind clinicians | Development of an online decision tool integrated into the primary care EHR system |
| Before trial (Apr 2024-Dec 2024) | **1B, 1C, 1D, 1F, 1G, 2D, 3A, 3E, 3F, 4A** | Inform local opinion leaders  Build a coalition | Inform secondary care specialists ((paediatric) surgeons, paediatricians and emergency department physicians) about the study when deemed necessary, that is, in case of frequent CRP POCT in high-risk patients at the request of the secondary care specialist |
| During trial  (Jan 2025-Mar 2028) | **1B, 1C, 1D, 3I, 4A** | Conduct educational outreach visits | Kick-off visits to general practices |

Abbreviations: GP, general practitioner; EHR; electronic health record; CRP, C-reactive protein; POCT, point-of-care testing

**Supplementary 5. Summary of e-learning modules**

The **Introduction module** covers acute abdominal pain and appendicitis in children, including diagnostic recommendations from the Dutch GP guideline.

In the **Clinical Prediction Rule module**, GPs learn about the use of clinical prediction rules in general, the prediction rule for appendicitis in primary care, and the diagnostic strategy with management advice per risk group. This module includes interactive questions based on a video case involving a high-risk child.

The **CRP module** presents a medium-risk case and explains when CRP testing is indicated, how to interpret results, and how symptom duration and cut-off values inform clinical decisions.

The **Communication module** introduces a low-risk case in which referral is requested, and offers strategies for discussing management options with parents and children across risk groups.

The **Final Test module** concludes the training with ten questions assessing understanding of the diagnostic strategy.

**Supplementary 6. Case report form**

**INCLUSION FORM INTERVENTION GROUP**

**Inclusion criteria:**

The child is in the age group 4 to 18 years.	Yes/No

The child has acute abdominal pain (duration max. 7 days).	Yes/No

**Exclusion criteria:**

The child has undergone an appendectomy.	Yes/No

The child is pregnant.	Yes/No

The abdominal pain is caused by trauma.	Yes/No

**Prediction rule:**

1. Male sex	Yes/No

2. Nausea of vomiting	Yes/No

3. Duration of abdominal pain	< 24 hours / 24-48 hours / > 48 hours

4. Elevated body temperature (≥ 37.3 °C)	Yes/No
*This feature is considered present if a temperature of
≥ 37.3 °C was measured rectally or in the ear, either by the
GP or at home.*

5. Abnormal bowel sounds (ileus peristalsis)	Yes/No
*Absent, tinkling, or high-pitched bowel sounds*

6. Tenderness on palpation of the right lower quadrant	Yes/No

7. Peritoneal irritation	Yes/No
*Present if one or more of the following findings are observed:
muscle guarding, active muscular resistance, rebound
tenderness, or pain with jarring movements (e.g., pain when
jumping, pain during transport).*

**Score prediction rule (0-17)**

**Risk group of child and management advice**

- Low risk of appendicitis (0.5%): Consider another diagnosis and provide safety-netting advice.
- Medium risk of appendicitis (7.5%): Perform CRP POCT
- CRP <10 mg/L: low risk of appendicitis (probability 1.3%). Consider another diagnosis and provide safety-netting advice.
- CRP 10-50 mg/L: medium risk of appendicitis (probability 16.8%). Perform a reassessment with CRP POCT if necessary **or** Refer the child to the Emergency Department.
- CRP >50 mg/L: high risk of appendicitis (probability 41.8%). Refer the child to the Emergency Department.
- No CRP POCT performed: No referral advice can be given.
- High risk of appendicitis (probability 41.0%): Refer the child to the Emergency Department.

**Brief explanation of the study for parents/child:**

A study is currently being conducted on the use of a decision-making tool for children with acute abdominal pain. Your child is eligible to participate. For this study, you (and your child) will be asked to complete questionnaires.

Is it okay if I share your contact details (name, email address, and phone number) and information about this visit with the researchers at the UMCG? They will then send you more information about the study by email.

**Does the parent/child give permission to share the relevant information with the researchers?** *Note: Please also document the consent or objection, and the reason for it, in the child’s medical record.*

- Yes/No

**Additional information**

Name of GP	……..

Date of visit	……..

Name of child	……..

Age of child	……..

Email address of parent/child	……..

Phone number of parent/child	……..

**Process evaluation**

Did you perform CRP POCT?	Yes/No

Why did you (not) perform CRP POCT?	……..

Did you refer the child to the hospital?	Yes/No

Why did you (not) refer the child?	……..

**INCLUSION FORM CONTROL GROUP**

**Inclusion criteria:**

The child is in the age group 4 to 18 years.	Yes/No

The child has acute abdominal pain (duration max. 7 days).	Yes/No

**Exclusion criteria:**

The child has undergone an appendectomy.	Yes/No

The child is pregnant.	Yes/No

**Brief explanation of the study for parents/child:**

A study is currently being conducted on the use of a decision-making tool for children with acute abdominal pain. Your child is eligible to participate. For this study, you (and your child) will be asked to complete questionnaires.

Is it okay if I share your contact details (name, email address, and phone number) and information about this visit with the researchers at the UMCG? They will then send you more information about the study by email.

**Does the parent/child give permission to share the relevant information with the researchers?** *Note: Please also document the consent or objection, and the reason for it, in the child’s medical record.*

- Yes/No

**Additional information**

Name of GP	……..

Date of visit	……..

Name of child	……..

Age of child	……..

Email address of parent/child	……..

Phone number of parent/child	……..

**Process evaluation**

Did you perform CRP POCT?	Yes/No

Why did you (not) perform CRP POCT?	……..

Did you refer the child to the hospital?	Yes/No

Why did you (not) refer the child?	……..

**Supplementary 7: Data collection from medical records over 3 months**

| **Variable** |  |  |  |  |
| --- | --- | --- | --- | --- |
| **Symptoms during initial consultation** |  |  |  |  |
| Nausea or vomiting | 1. Not reported 2. Yes 3. No |  |  |  |
| Duration of abdominal pain | 1. Not reported 2. Less than 24 hours 3. Between 24 and 48 hours 4. More than 48 hours |  |  |  |
| Elevated body temperature | 1. Not reported 2. Yes, temperature measured and was… 3. Elevated temperature reported, but temperature not measured 4. No, temperature measured and was … 5. Absence of elevated temperature reported, temperature not measured |  |  |  |
| Abnormal bowel sounds (absent, tinkling or high-pitched) | 1. Not reported 2. Yes 3. No |  |  |  |
| Palpation pain right lower quadrant | 1. Not reported 2. Yes 3. No |  |  |  |
| Peritoneal irritation (abdominal rigidity, guarding, rebound tenderness and/or pain with jarring movements) | 1. Not reported 2. Yes 3. No | Specify:   1. Abdominal rigidity 2. Guarding 3. Rebound tenderness 4. Pain with jarring movements |  |  |
| Anorexia (lack of appetite) | 1. Not reported 2. Yes 3. No |  |  |  |
| Other symptoms related to abdominal pain | 1. Yes 2. No | Specify symptoms *(free text)* |  |  |
| **Diagnostic tests by GP** |  |  |  |  |
| CRP POCT | 1. Yes 2. No | Test result |  |  |
| *Venous blood test* |  |  |  |  |
| Haemoglobin | 1. Yes 2. No | Test result |  |  |
| Leucocytes | 1. Yes 2. No | Test result |  |  |
| Neutrophils | 1. Yes 2. No | Test result |  |  |
| Neutrophiles percentage | 1. Yes 2. No | Test result |  |  |
| Thrombocytes | 1. Yes 2. No | Test result |  |  |
| CRP | 1. Yes 2. No | Test result |  |  |
| ESR | 1. Yes 2. No | Test result |  |  |
| *Faeces test* |  |  |  |  |
| Faecal culture | 1. Yes 2. No | Test result |  |  |
| Faecal ova and parasite test | 1. Yes 2. No | Test result |  |  |
| *Urine test* |  |  |  |  |
| Urine stick | 1. Yes 2. No | Test result |  |  |
| Urine dipslide | 1. Yes 2. No | Test result |  |  |
| Urine culture | 1. Yes 2. No | Test result |  |  |
| *Imaging* |  |  |  |  |
| Abdominal ultrasound | 1. Yes 2. No | Test result |  |  |
| CT abdomen | 1. Yes 2. No | Test result |  |  |
| MRI abdomen | 1. Yes 2. No | Test result |  |  |
| *Other diagnostic test* | 1. Yes 2. No | Specify test and result *(free text)* |  |  |
| **Diagnosis GP** |  |  |  |  |
| GP’s diagnosis at initial consultation | 1. Appendicitis 2. Non-specific abdominal pain 3. Constipation 4. Gastro-enteritis 5. Urinary tract infection 6. Pyelonephritis 7. PID 8. Inguinal hernia 9. Mesenteric lymphadenitis 10. Testicular torsion 11. Ovarian torsion 12. FGID 13. IBD 14. Other, namely: …. |  |  |  |
| GP’s diagnosis at 3 months follow-up | 1. Appendicitis 2. Non-specific abdominal pain 3. Constipation 4. Gastro-enteritis 5. Urinary tract infection 6. Pyelonephritis 7. PID 8. Inguinal hernia 9. Mesenteric lymphadenitis 10. Testicular torsion 11. Ovarian torsion 12. FGID 13. IBD 14. Other, namely: …. |  |  |  |
| **Management GP** |  |  |  |  |
| Medication prescribed by GP | 1. Yes 2. No | Type of medication:   1. Name 2. Dosage 3. Frequency 4. Duration |  |  |
| Referral to hospital | 1. Yes 2. No | Medical specialist:   1. Emergency department 2. (Paediatric) surgeon 3. Paediatrician 4. Paediatric gastroenterologist 5. Gynaecologist 6. Internal medicine specialist 7. Gastroenterologist 8. Other, namely … |  |  |
| Reason for referral according to GP | *Free text* |  |  |  |
| Planned reassessment | 1. Yes, physical reassessment 2. Yes, reassessment by phone contact 3. No |  |  |  |
| **Diagnosis hospital** |  |  |  |  |
| Final diagnosis based on latest hospital correspondence | 1. Appendicitis 2. Non-specific abdominal pain 3. Constipation 4. Gastro-enteritis 5. Urinary tract infection 6. Pyelonephritis 7. PID 8. Inguinal hernia 9. Mesenteric lymphadenitis 10. Testicular torsion 11. Ovarian torsion 12. FGID 13. IBD 14. Other, namely: …. |  |  |  |
| *Appendicitis diagnosis* |  |  |  |  |
| Simple or complex appendicitis (based on: presence of necrosis, perforation, abscess) | 1. Not reported 2. Simple appendicitis 3. Complex appendicitis |  |  |  |
| Method for classification into simple or complex appendicitis | 1. Not reported 2. Intraoperative findings 3. Imaging findings 4. Other, namely: … |  |  |  |
| Type of appendicitis based on histopathology | 1. Not reported 2. Phlegmonous 3. Gangrenous 4. Perforated 5. Other, namely …. |  |  |  |
| Presence of neuroendocrine tumor in appendix | 1. Yes 2. No |  |  |  |
| **Diagnostic tests hospital** |  |  |  |  |
| CRP POCT | 1. Yes 2. No | Test result |  |  |
| *Venous blood test* |  |  |  |  |
| Haemoglobin | 1. Yes 2. No | Test result |  |  |
| Leucocytes | 1. Yes 2. No | Test result |  |  |
| Neutrophils | 1. Yes 2. No | Test result |  |  |
| Neutrophiles percentage | 1. Yes 2. No | Test result |  |  |
| Thrombocytes | 1. Yes 2. No | Test result |  |  |
| CRP | 1. Yes 2. No | Test result |  |  |
| ESR | 1. Yes 2. No | Test result |  |  |
| *Faeces test* |  |  |  |  |
| Faecal culture | 1. Yes 2. No | Test result |  |  |
| Faecal ova and parasite test | 1. Yes 2. No | Test result |  |  |
| *Urine test* |  |  |  |  |
| Urine stick | 1. Yes 2. No | Test result |  |  |
| Urine dipslide | 1. Yes 2. No | Test result |  |  |
| Urine culture | 1. Yes 2. No | Test result |  |  |
| *Imaging* |  |  |  |  |
| Abdominal ultrasound | 1. Yes 2. No | Test result |  |  |
| CT abdomen | 1. Yes 2. No | Test result |  |  |
| MRI abdomen | 1. Yes 2. No | Test result |  |  |
| *Other diagnostic test* | 1. Yes 2. No | Specify test and result *(free text)* |  |  |
| **Management hospital** |  |  |  |  |
| Hospital admission | 1. Yes 2. No |  |  |  |
| Duration of hospital admission in days | *Free text* |  |  |  |
| Surgery | 1. Yes 2. No | Type of surgery:   1. Not reported 2. Laparoscopic appendectomy 3. Open appendectomy 4. Other, namely … | Presence of postoperative complications:   1. Not reported 2. Yes, namely …. 3. No | Re-intervention performed:   1. Not reported 2. Yes, namely … 3. No |
| Medication prescribed by hospital | 1. Not reported 2. Yes 3. No | Specify type of medication and other information *(free text)* |  |  |
| Other treatment in hospital | 1. Yes 2. No | Specify treatment *(free text)* |  |  |
| **Adverse outcomes** |  |  |  |  |
| Adverse outcomes of appendicitis (peritonitis, sepsis, ileus, other) | 1. Not reported 2. Yes 3. No | Specify:   1. Peritonitis 2. Sepsis 3. Ileus 4. Other, namely … |  |  |
| Adverse outcomes related to diagnosis other than appendicitis | 1. Yes 2. No | Specify adverse outcome *(free text)* |  |  |
| Death of child | 1. Yes 2. No | Specify cause of death *(free text)* |  |  |
| **Healthcare use** |  |  |  |  |
| Number of GP consultations | Number from 1-5 |  |  |  |
| GP out-of-hours service | 1. Yes 2. No | How often |  |  |
| Emergency department | 1. Yes 2. No | How often |  |  |
| General surgeon | 1. Yes 2. No | How often |  |  |
| Paediatric surgeon | 1. Yes 2. No | How often |  |  |
| Other specialist | 1. Yes, namely… 2. No | How often |  |  |

Abbreviations: GP, general practitioner; CRP, C-reactive protein; POCT, point-of-care test; ESR, erythrocyte sedimentation rate; PID, pelvic inflammatory disease; FGID, functional gastrointestinal disease; IBD, inflammatory bowel disease

**Supplementary 8. Patient participation matrix**
